# First investigation of blood parasites of bats in Burkina Faso detects *Hepatocystis* parasites and infections with diverse *Trypanosoma* spp.

**DOI:** 10.1007/s00436-023-08002-2

**Published:** 2023-10-17

**Authors:** Noel Gabiliga Thiombiano, Magloire Boungou, Bertrand Adéchègoun Mèschac Chabi, Adama Oueda, Oskar Werb, Juliane Schaer

**Affiliations:** 1 Laboratoire de Biologie et Ecologie Animales (LBEA), Unite de Formation Et de Recherche/Science de La Vie et de La Terre (UFR/SVT), University Joseph KI-ZERBO, Ouagadougou, Burkina Faso; 2 Universite de Ouahigouya, Ouahigouya, Burkina Faso; 3 Department of Molecular Parasitology, Institute of Biology, Humboldt University, Berlin, Germany

**Keywords:** *Hepatocystis*, *Trypanosoma*, Bats, Haemosporida

## Abstract

Bats are hosts to a large diversity of eukaryotic protozoan blood parasites that comprise species of *Trypanosoma* and different haemosporidian parasite taxa and bats have played an important role in the evolutionary history of both parasite groups. However, bats in several geographical areas have not been investigated, including in Burkina Faso, where no information about malaria parasites and trypanosomes of bats exists to date.

In this study, we collected data on the prevalence and the phylogenetic relationships of protozoan blood parasites in nine different bat species in Burkina Faso. *Hepatocystis* parasites were detected in two species of epauletted fruit bats, and a relatively high diversity of trypanosome parasites was identified in five bat species. The phylogenetic analyses recovered the trypanosome parasites of the bat species *Rhinolophus alcyone* and *Nycteris hispida* as close relatives of *T. livingstonei*, the trypanosome infections in *Scotophilus leucogaster* as closely related to the species *T. vespertilionis* and the trypanosomes from *Pipistrellus nanulus* and *Epomophorus gambianus* might present the species *T. dionisii.* These findings of the first investigation in Burkina Faso present a first snapshot of the diversity of protozoan blood parasites in bats in this country.

**Supplementary Information:**

The online version contains supplementary material available at 10.1007/s00436-023-08002-2.

## Introduction

Bats (order Chiroptera) represent up to 20% of all mammalian species with 1470 species described to date (Simmons and Cirranello 2023). They are considered key organisms in maintaining the ecosystem balance as they play essential roles in regulating insect populations, pollination, and seed dispersal (e.g., Kalka et al. 2008; Kunz et al. 2011; Aziz et al. 2021). Bats are hosts to a large diversity of eukaryotic protozoan blood parasites that comprise species of *Trypanosoma* and different haemosporidian parasite taxa and several studies suggest that bats have played an important role in the evolutionary history of both parasite groups (e.g., Duval et al. 2007; Hamilton et al. 2012; Lima et al. 2012, 2013; Schaer et al. 2013; Perkins and Schaer 2016; Galen et al. 2018; Clement et al. 2020; Austen and Barbosa 2021).

Trypanosomes (Kinetoplastea: Trypanosomatidae) are protozoan blood parasites that are distributed across all continents. They have adapted to infect all classes of vertebrates, feature complex life cycles and are primarily transmitted to vertebrate hosts through blood-feeding arthropods or leeches (e.g., Simpson et al. 2006). Different *Trypanosoma* species pose threats to both humans and livestock; in Africa these comprise the three *Trypanosoma* species *Trypanosoma brucei *sensu lato, *Trypanosoma vivax* and *Trypanosoma congolense* (Morrison et al. 2016; Büscher et al. 2017). Bats are known to harbor a wide range of *Trypanosoma* species; however, our understanding of species diversity, vectors, life cycles, distribution, and the evolutionary history of bat trypanosomes remains limited (e.g., Hamilton et al. 2012; Lima et al. 2012, 2013; Clement et al. 2020; Austen and Barbosa 2021).

Haemosporidian parasites, belonging to the phylum Apicomplexa, infect a wide array of birds, saurian reptiles, and mammals (Garnham 1966). The *Plasmodium* species that infect humans and cause the malaria disease are part of a large group of haemosporidian parasites, encompassing approximately 500 closely related species (Martinsen and Perkins 2013; Galen et al. 2018). These parasites utilize a diverse range of dipteran and vertebrate hosts to complete their life cycles (Garnham 1966; Levine 1988). Mammalian haemosporidian parasites are classified into different genera, including *Plasmodium*, *Hepatocystis*, *Polychromophilus* and *Nycteria* (Perkins and Schaer 2016). Among mammals, bats seem to feature an exceptionally high diversity of haemosporidian parasites and comprehensive sampling and systematic analysis of bat malaria parasites are vital for gaining a better understanding of the evolutionary history of haemosporidian parasites, including those that infect humans (e.g., Garnham 1966; Schaer et al. 2013; Perkins and Schaer 2016).

Despite the large haemosporidian and trypanosome parasite diversity being reported from bats, data from several geographical areas have not been collected yet. One of these “blind spots” is in Burkina Faso where no information about haemosporidian parasites and trypanosomes of bats exists to date. With about 52 species of bats, Burkina Faso features a diverse fauna of bats (Kangoyé et al. 2012, 2015; Thiombiano et al. 2019).

This study presents data on the prevalence and the phylogenetic relationships of protozoan blood parasites in different bat species in Burkina Faso.

## Materials and methods

### Field sampling and microscopy

**Fig. 1:**
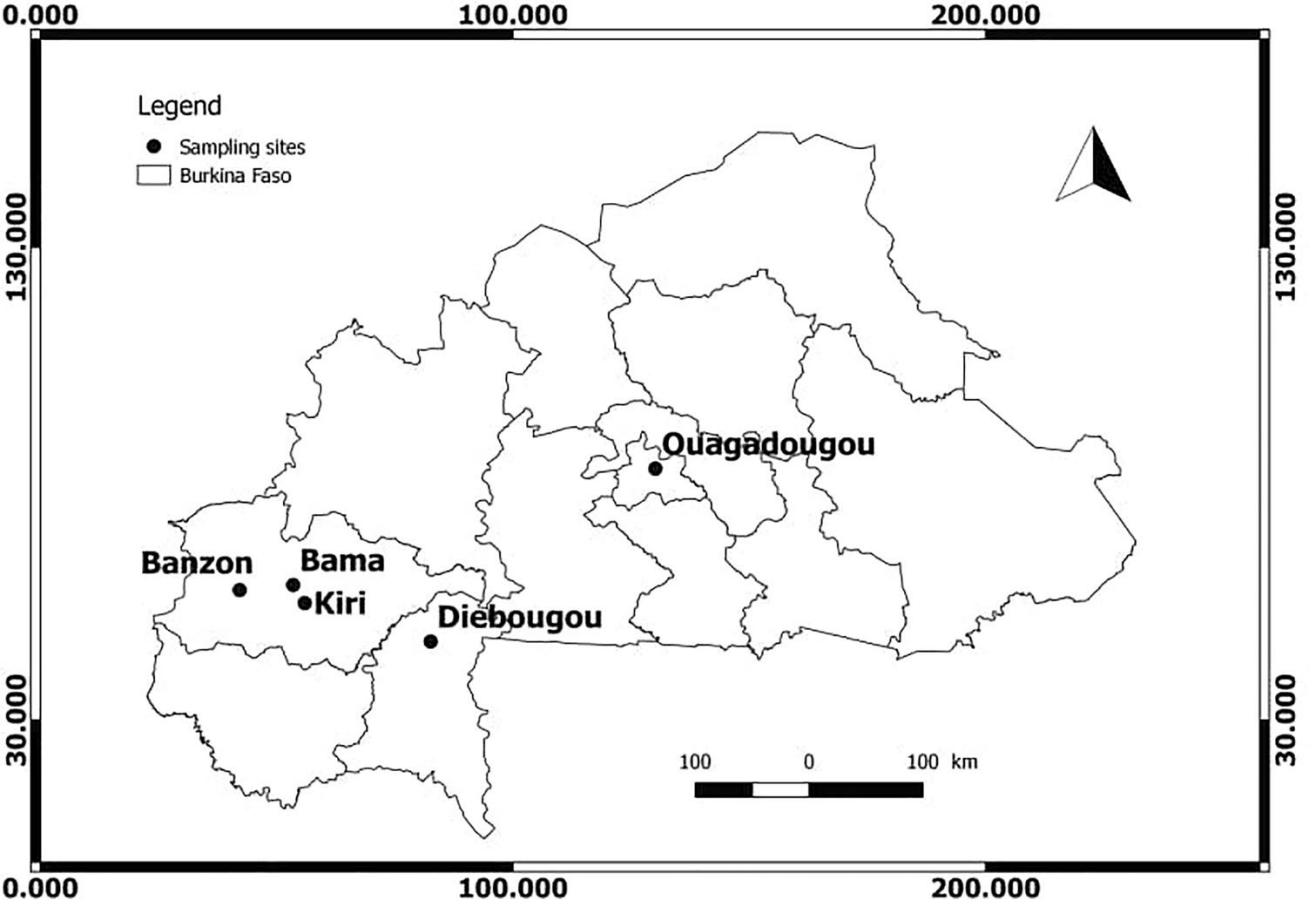
**Map of Burkina Faso.** Sampling sites are depicted by dots. The sampling sites Bama, Banzon, Kiri and Ouagadougou are characterized by the presence of fruit tree orchards (e.g., Mango trees), while the sampling site in Diebougou is located close to a cave

Bats were sampled in Burkina Faso in December 2020 (wet season), January to April 2021 (dry season) and August 2021 (wet season) (Table S1). Burkina Faso is a Sahelian country located in West Africa. Its population was estimated at 21,509,443 in 2021 (INSD, 2021). Burkina Faso is characterized by a tropical climate alternating between a long dry season (October to April) and a short rainy season (May to September). Sampling was carried out at five different sampling sites that were characterized by the presence of fruit tree orchards (e.g., Mango trees), and only the sampling site in Diebougou was located close to a cave (Fig. 1).

Bats were captured using mist nets. The nets were set every capture night between 5 pm and 5 am. The bats were gently removed from the nets and transferred into individual bags. Measurements were taken to identify the bats to genus and/or species level using different identification keys (Rosevear 1965; Hayman 1971; Bergmans 1997). For the investigation of blood parasites, small blood samples were collected by venous puncture of the brachial vein and collected in capillary tubes. These blood samples were used to spot blood dots onto DNA FTA cards for subsequent molecular analysis and to prepare thin blood smears on slides for microscopic analysis. All bats were released at the capture sites, and it was assured that the bleeding had stopped. The thin blood smears were dried and fixed in 99–100% (vol/vol) ethanol solution for 3 s in the field and fixed again with 100% (vol/vol) methanol in the laboratory (in Germany), before staining with 10% Giemsa solution for about 40 min. Giemsa-stained blood smears were examined for the presence of haemosporidian and trypanosome parasites using light microscopy at a magnification of × 400 and × 1000 with immersion oil.

### Molecular methods and phylogenetic analysis

Whole genomic DNA was extracted from the dried blood dots on DNA FTA cards using the DNeasy extraction kit (Qiagen, Hilden, Germany) (e.g., Schaer et al. 2017). The protocol for animal tissues was performed and samples were eluted in 80–100 µl AE buffer. PCRs were performed using the AllTa q Master Mix Kit (QIAGEN) with 4–5 µl of genomic DNA as the template, and 1 µl of each primer (10 mM). For the detection and phylogenetic analysis of haemosporidian parasites (*Plasmodium*, *Hepatocystis*, *Nycteria*, *Polychromophilus*), the mitochondrial genes cytochrome b (*cytb*) and cytochrome oxidase 1 (*cox1*) and the nuclear gene elongation factor 2 (*ef2*) of the parasites were amplified and Sanger-sequenced (following e.g., Schaer et al. 2013). To detect and characterize trypanosome infections, a nested-PCR approach was employed and about 640 bp of the small subunit 18S ribosomal RNA gene (*18S rRNA*) was amplified (using the approach described by Noyes et al. 1999). Following this, for all the samples that tested positive, a second gene, the nuclear glycosomal glyceraldehyde phosphate dehydrogenase (*gGAPDH*), was targeted using a nested-PCR approach (as outlined by Clement et al. 2020). All primers are listed in Table S2. All positive PCR products were sequenced with the amplification primers and run on an ABI-373 sequencer. The software programs *Geneious Prime 2023.1.2* (https://www.geneious.com) and *MEGA 11.0.10* (https://www.megasoftware.net) were used to quality-check and manually edit all nucleotide sequences. Instances of double nucleotide peaks (where both peaks have a minimum height of 40% at a single nucleotide position) observed in the sequence electropherograms of sequence segments with high quality within individual sequence assemblies were documented as indicative of mixed haplotype infections. Amplification and sequencing were repeated for samples with sequences of lower quality. Individual sequences per sample were assembled and exported as a consensus sequence for subsequent analysis. Sequences were aligned using the MAFFT algorithm (Katoh et al. 2002; Katoh and Standley 2013). GenBank was utilized to obtain reference sequences, which were subsequently added to the alignments. Accession numbers for the analysis of haemosporidian parasites are provided in Table S3, while the accession numbers for the 18S rRNA and *gGAPDH* trypanosome analyses are provided in the respective phylogenetic tree figures and Table S4. The concatenated alignment of three genes for the analysis of the phylogenetic relationships of haemosporidian parasites comprised 123 sequences (including sequences from 16 representative samples of this study) and had a total length of 1938 nucleotides (nt) (414 nt of *ef2* gene, 531 nt of *cytb*, 993 nt of *cox1*). The alignment of *cytb* for phylogenetic analysis of *Hepatocystis* parasites in African bats comprised 82 sequences and had a length of 531 nt. Sequence alignments for the analysis of *Trypanosoma* spp. included 89 sequences and a length of 726 nt for *18S rRNA* and 79 sequences and 894 nt for *gGAPDH* respectively. Data was partitioned according to genes (for the protein-coding genes) and the software *modeltest-ng 0.1.7* (Darriba et al. 2020), implemented in *raxmlGUI version 2.0.10* (Edler et al. 2021) was used to test different DNA substitution models. Maximum Likelihood (ML) analyses were conducted using *raxmlGUI 2.0.10* to evaluate phylogenetic relationships. The haemosporidian parasite analysis of the concatenated dataset was carried out using the substitution model GTR + I (proportion of invariant) + Gamma (rate heterogeneity) and the taxon *Leucocytozoon* as outgroup and the nodal support was evaluated using 1000 replicates (thorough bootstrap). The *Hepatocystis* parasite analysis of the *cytb* dataset was carried out using the substitution model TIM2 + I (proportion of invariant) and the taxon *Plasmodium falciparum* as outgroup and the nodal support was evaluated using 1000 replicates (thorough bootstrap). The Maximum Likelihood analyses of the trypanosome datasets of the *18S rRNA* and *gGAPDH* gene were carried out using the models TIM3 + I + G and GTR + I + G (with 10,000 thorough bootstrapping) respectively and the outgroup taxon *Trypanosoma lewisi* (following Kamani et al. 2022). All resulting phylogenetic trees were displayed in FigTree v1.4.4 (http://tree.bio.ed.ac.uk/software/figtree/).

## Results

### Prevalence of protozoan blood parasites

A total of 119 bats belonging to six bat families, seven genera and nine species were investigated. Screening with microscopy and PCR methods detected haemosporidian and trypanosome parasite infections in bats of Burkina Faso (Table 1**; **Table S1). Haemosporidian parasites were recorded in 26 bat individuals (26/119), corresponding to a prevalence of 21.8%. These parasites, identified as species of the genus *Hepatocystis*, were detected in individuals of the two bat species *Epomophorus gambianus* and *Epomophorus pusillus* (bat family Pteropodidae) that were sampled in the two locations of Bama and Ouagadougou. All ten *E. pusillus* individuals were captured in the wet season and featured infections with *Hepatocystis* parasites (10/10, 100%). The prevalence of infections in *E. gambianus* was 41,1% (16/38), with 10% (4/38) in samples from the dry season and a higher prevalence of 32% (12/38) in the individuals that got captured in the wet season. Although previous research has identified *Nycteria* and *Polychromophilus* parasites in bat species belonging to the Nycteridae, Rhinolophidae, and Vespertilionidae, we did not detect haemosporidian infections in bats of these bat families in this study (Table 1).

**Table 1:** Investigated bat species and the prevalence of protozoan blood parasites

Bat family	Bat species	Prevalence Haemosporida in %^1^	Haemosporidian taxon	Prevalence of *Trypanosoma* in %^1^	*Trypanosoma* taxon^2^
*Pteropodidae*	*Epomophorus gambianus*	42.1 (16/38)	*Hepatocystis* sp.	2.6 (1/38)	*T.* sp. (*T*. cf. *dionisii*)
	*Epomophorus pusillus*	100 (10/10)	*Hepatocystis* sp.	0 (0/10)	–-
*Hipposideridae*	*Hipposideros jonesi*	0 (0/1)	–-	0 (0/1)	–-
*Molossidae*	*Mops condylurus*	0 (0/3)	–-	0 (0/3)	–-
	*Mops midas*	0 (0/16)	–-	0 (0/16)	–-
*Nycteridae*	*Nycteris hispida*	0 (0/1)	–-	100 (1/1)	*T.* sp. (*T*. cf. *livingstonei*)
*Rhinolophidae*	*Rhinolophus alcyone*	0 (0/11)	–-	36.4 (4/11)	*T.* sp. (*T*. cf. *livingstonei*); *T.* sp.
*Vespertilionidae*	*Pipistrellus nanulus*	0 (0/10)	–-	10.0 (1/10)	*T.* sp. (*T*. cf. *dionisii*)
	*Scotophilus leucogaster*	0 (0/25)	–-	17.2 (5/29)	*T.* sp. (*T*. cf. *vespertilionis*)
Total		21.8% (26/119)		10.1% (12/119)	

^1^Number of infected individuals/total individuals are given in parenthesis, ^2^tentative ID based on topology/phylogenetic relationships recovered in the 18S rRNA and *gGAPDH* phylogenetic analyses

*Trypanosoma* spp. parasites were detected in 12 individuals (12/119) corresponding to an overall prevalence of 10.1% in individuals of five different bat species belonging to four bat families captured in the three locations Bazon, Diebougou and Ouagadougou (Table 1). Trypanosome infections were recorded in one individual per species in *Epomophorus gambianus* (1/38 = 2.6%), *Nycteris hispida* (1/1 = 100%) and *Pipistrellus nanulus* (1/10 = 10%)*.* Prevalences of 36% and 17% were recorded for *Rhinolophus alcyone* (4/11 = 36.4%) and *Scotophilus leucogaster* (5/29 = 17.2%) respectively*.*

### Characterization of *Hepatocystis* parasites of bats in Burkina Faso

**Fig. 2:**
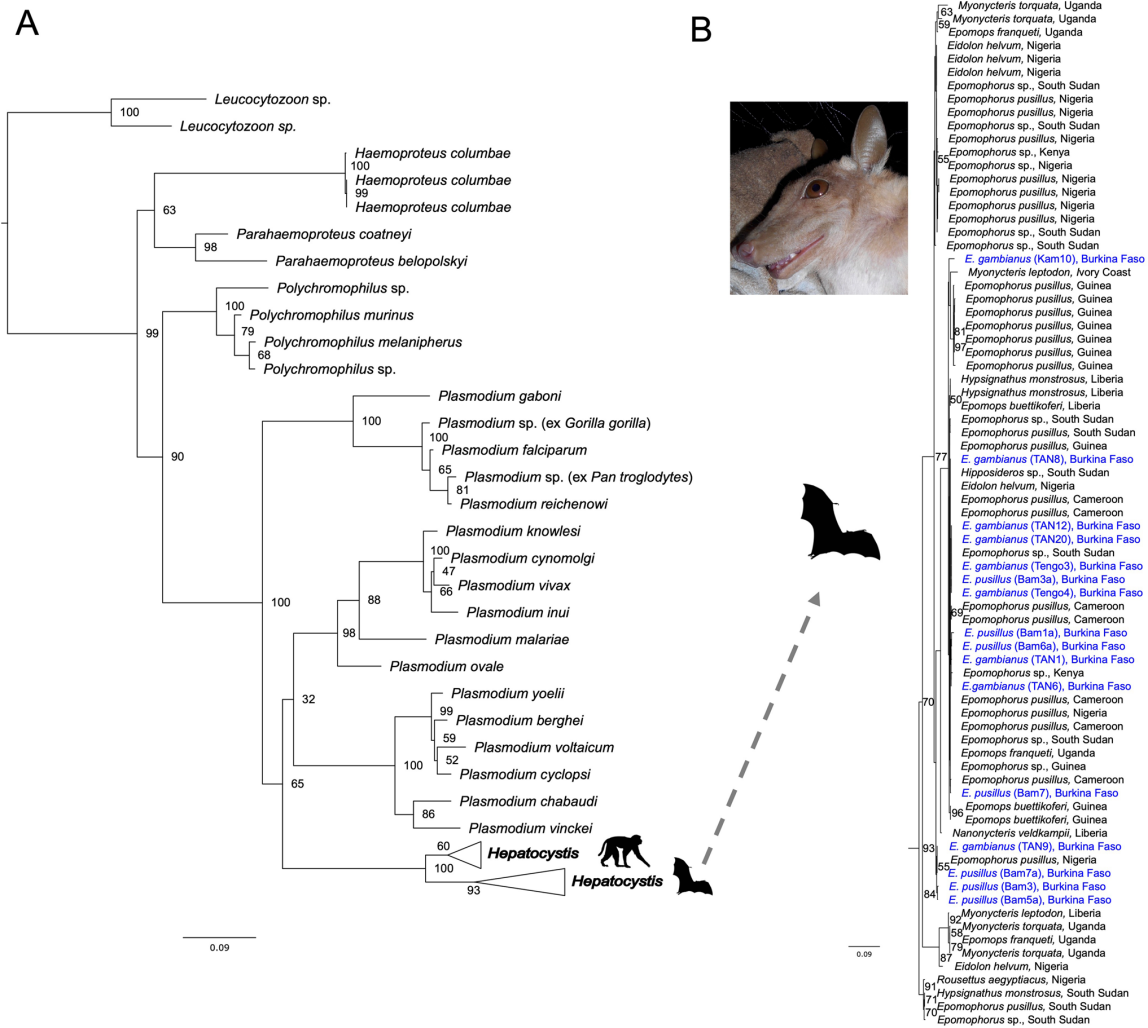
**Maximum likelihood analysis of *Hepatocystis* parasites in African bat species**. **A** The analysis is based on the concatenated dataset of the parasites mitochondrial genes *cytb* (531nt) and cox1 (993nt) and one nuclear gene (414 nt of *ef2* gene) and was run in the context of the major haemosporidian parasite clades *Haemoproteus*, *Parahaemoproteus*, *Polychromophilus* and the mammalian-infecting *Plasmodium* clades. *Leucocytozoon* was used as outgroup taxon. The phylogenetic analysis recovered the *Hepatocystis* sequences from *E. pusillus* and *E. gambianus* of this study within the African bat *Hepatocystis* clade (collapsed in A). **B** Section of the African bat *Hepatocystis* clade (uncollapsed). No strict clustering of African bat *Hepatocystis* sequences according to country or host species is apparent which is in line with previous findings (e.g., Schaer et al. 2017). Sequences of the study from Burkina Faso are highlighted in blue. Numbers at nodes are ML bootstrap values (> 50) using 1000 replicates. The photograph depicts the bat host species *E. gambianus*

The quality of the blood smears did not permit a detailed evaluation of the morphology of the gametocyte blood stages of the *Hepatocystis* parasites of *E. pusillus* and *E. gambianus*. However, as characteristic for *Hepatocystis* parasites, the detected blood stages were limited to gametocyte stages (mostly mature stages) and hemozoin pigment was present in all parasite cells in the infected erythrocytes (e.g., Garnham 1966; Schaer et al. 2013; Atama et al. 2019) (Fig. S1). High quality sequences for at least one molecular marker could be generated for 25 of the 26 samples. For one sample (isolate Tengo1) only a low-quality sequence for the partial cox1 gene was available that however shared highest identity with *Hepatocystis* sp. of African bats in NCBI BLASTn search (e.g., with NCBI accession number MZ460952) and was thus scored as infected with *Hepatocystis* sp. All *Hepatocystis* infections in *E. pusillus* and *E. gambianus* of this study were determined as mixed haplotype infections as double nucleotide peaks were recorded in the sequences of all samples. As haplotype network analysis can only be performed with single haplotype infections, the number of haplotypes present in our sample set could not be determined. However, the phylogenetic analysis confirmed the parasites of *E. pusillus* und *E. gambianus* from Burkina Faso as *Hepatocystis* sp. which group in the main *Hepatocystis* clade that contains all other African bat *Hepatocystis* parasites (Fig. 2). Within this clade, the *Hepatocystis* parasite sequences of this study (highlighted in blue) fall in different places and do not cluster in host genus or species-specific clades just like the bat *Hepatocystis* sequences from other countries and locations in both West Africa and Central-/East Africa that exhibit no pattern of clustering according to countries or host species (Fig. 2, Fig. S2).

### Molecular characterization of *Trypanosoma *spp. parasites of bats in Burkina Faso

**Fig. 3:**
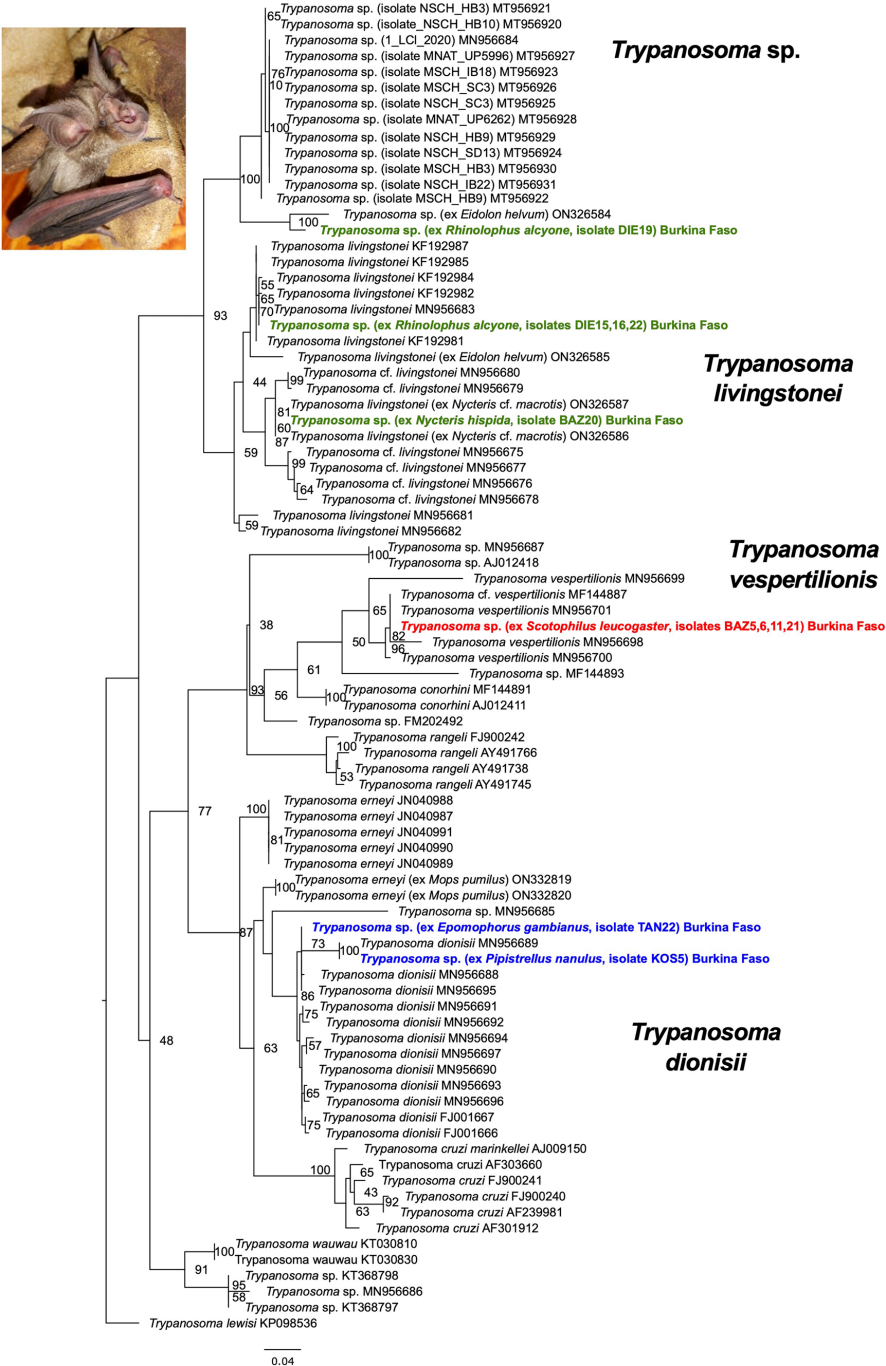
**Maximum likelihood phylogeny of the *Trypanosoma* parasites based on the 18S rRNA alignment.** The alignment featured a total length of 726 bp and the evolutionary model TIM3 + I + G was used for the phylogenetic analysis and the taxon *Trypanosoma lewisi* as outgroup. Numbers at nodes are ML bootstrap values using 10,000 replicates. The representative sequence for the haplotype 1 recovered in three *R. alcyone* samples groups within the *T. livingstonei* clade, the sequence of the fourth trypanosome sample from *R. alcyone* of this study (isolate DIE19) features highest sequence similarity with a *Trypanosoma* sp. from *Eidolon helvum* in Nigeria. The trypanosome parasite detected in *N. hispida* shares highest sequence identity with a trypanosome isolated from *Nycteris macrotis* from Nigeria which together group within the *T. livingstonei*/*Trypanosoma *cf.* livingstonei* clade. All trypanosome sequences of *R. alcyone* and *N. hispida* are highlighted in bold green. The trypanosome sequences from the parasites isolated from *S. leucogaster* (highlighted in bold red) group in a clade that comprises *Trypanosoma vespertilionis* sequences. The trypanosome sequences from *P. nanulus* and *E. gambianus* group with *T. dionisii* sequences recovered from diverse bat species (the *gGAPDH* sequences for both samples group within the *T. dionisii* clade with high support, see Fig. S3). The photograph depicts the bat host species *R. alcyone*

Three of the four trypanosome samples of *R. alcyone* (Rhinolophidae) share identical 18S rRNA sequences and thus represent one haplotype (isolates DIE15, DIE16, DIE22). The 18S rRNA phylogenetic analysis recovered this haplotype within the *T. livingstonei* clade*.* Close relatives are *T. livingstonei* parasites that were isolated from different bat species in Africa (*Hipposideros caffer*, Mozambique, KF192984; *Rhinolophus landeri*, Mozambique, KF192982 and *Rhinolophus simulator*, South Africa, MN956683) (Fig. 3). The sequence of the fourth trypanosome sample from *R. alcyone* of this study (isolate DIE19) features highest sequence similarity with a *Trypanosoma* sp. reported from *Eidolon helvum* in Nigeria. Together these two sequences group with a *Trypanosoma* sp. clade that comprises *Trypanosoma* parasite sequences from different *Miniopterus* bat hosts and bat flies from Europe and South Africa (Szentivanyi et al. 2020) and this whole clade shares a close relationship with *T. livingstonei* parasites (Fig. 3). The trypanosome parasite detected in *N. hispida* shares highest sequence identity with a reference sequence of a trypanosome isolated from *Nycteris macrotis* from Nigeria (ON326586) which together also group within the *T. livingstonei*/*Trypanosoma *cf*. livingstonei* clade. Unfortunately, no *gGAPDH* sequences could be successfully amplified for the trypanosomes of *R. alcyone* and *N. hispida*.

The trypanosome parasites in the five bat samples of *S. leucogaster* share highest identities with sequences of the species *Trypanosoma vespertilionis*. The 18S rRNA sequences of four samples (isolates BAZ5, BAZ6, BAZ11, BAZ21) represent one haplotype. The 18S rRNA phylogenetic analysis recovered this haplotype within *T. vespertilionis* sequences and with closest relationship with a *T. vespertilionis* reference of *Scotophilus* sp. from Guinea-Bissau (MF144889) (Fig. 3). The 18S rRNA of the fifth sample was of low quality. However, a high quality *gGAPDH* sequence for this sample was generated and the phylogenetic analysis confirmed this trypanosome sample of *S. leucogaster* (isolate BSOM18) as also belonging to the *T. vespertilionis*/*T.* cf. *vespertilionis* group (Fig. S3). The trypanosomes from *P. nanulus* and *E. gambianus* group with *T. dionisii* sequences from different bat species in the 18S rRNA analysis (Fig. 3) and further the *gGAPDH* sequences for both samples were recovered within the *T. dionisii* clade with high support (Fig. S3).

Unfortunately, no trypanosome parasite stages were detected in any of the blood smears of the bat hosts despite thorough screening, which points to very low parasitemia levels in all samples.

## Discussion

This study provides the first information on haemosporidian and trypanosome parasites of bats in Burkina Faso. *Hepatocystis* parasites were detected in two bat species, while trypanosomes were verified from five bat species. Interestingly, no co-infections of haemosporidian parasites and trypanosomes were detected in the study samples.

*Hepatocystis* parasites were detected in two species of epauletted fruit bats confirming previous findings of *Epomophorus* spp. being common *Hepatocystis* sp. hosts across their African distribution range (Schaer et al. 2013; Lutz et al. 2016; Boundenga et al. 2018; Atama et al. 2019). The morphospecies *Hepatocystis epomophori* has been described from *Epomophorus* species in Africa (Rodhain 1926), but it has been suggested that the *Hepatocystis* parasites of African epauletted fruit bats represent several close related taxa or cryptic species (Schaer et al. 2017). Unfortunately, the quality of the blood smears did not allow an assignment of the *Hepatocystis* parasites of the study to any morphospecies. However, the phylogenetic analyses recovered the parasites among sequences of parasites previously determined as belonging to a *Hepatocystis epomophori* species complex which lack signatures of host specificity and do not strictly group to country origin within Africa (Schaer et al. 2017). The recorded prevalences were comparable to findings of other studies in epauletted fruit bats in Africa (e.g., Schaer et al. 2013; Lutz et al. 2016; Boundenga et al. 2018) and we observed a seasonal trend with higher prevalence in the wet compared to the dry season samples confirming previous findings (Schaer et al. 2017). Interestingly, all *Hepatocystis* infections in this study represented mixed haplotype infections which might point to repeated infections over time with different parasite haplotypes that circulate in the area. Mixed haplotype infections have been reported before in *Hepatocystis* infections in primates and African epauletted fruit bats (Thurber et al. 2013; Schaer et al. 2017). Subsequent investigations are important to study the dynamics of *Hepatocystis* parasite infections in these bat hosts in Burkina Faso in more detail.

A relatively high diversity of trypanosome parasites was identified in the bat species of the study. Parasites that might represent *T. livingstonei* were recorded in the insectivorous bat species *R. alcyone* and *N. hispida*. Infections with *T. livingstonei* and *T*. cf. *livingstonei* seem to be prevalent in various African insectivorous bat species (Lima et al. 2013; Clement et al. 2020). The trypanosome infections in *S. leucogaster* feature closest phylogenetic relationships with the species *T. vespertilionis,* a species that has been reported from diverse European and African bats (Stevens et al. 1998; Espinosa-Álvarez et al. 2018; Clement et al. 2020). The trypanosomes from *P. nanulus* and *E. gambianus* share closest relationships with the species *T. dionisii*, a trypanosome species that is globally distributed and has been described in bats from the Americas, Europe, Africa and more recently also in Asian and Australian bats (e.g., Hamilton et al. 2012; Lima et al. 2013; Hodo et al. 2016; Espinosa-Álvarez et al. 2018; Mafie et al. 2018; Wang et al. 2019; Austen et al. 2020). These findings confirm the notion that African bat trypanosomes exhibit high phylogenetic diversity, potentially harboring a range of yet undiscovered species (Clement et al. 2020). Investigating the diversity and the phylogenetic relationships of bat trypanosomes is crucial for enhancing our understanding of the whole group of trypanosomes, as some species are a threat to humans (Hamilton et al. 2009; Lima et al. 2012). Consequently, a more targeted systematic sampling and subsequent molecular characterization of trypanosome species from African bats is needed.

The current study only represents a snapshot of the diversity of protozoan bat blood parasites in Burkina Faso as only nine of the 52 bat species of Burkina Faso have been investigated. Further investigations targeting a broader taxon sampling with larger sample sizes collected across different seasons will provide a more complete picture.

### Supplementary Information

Below is the link to the electronic supplementary material.

Supplementary file1 (JPG 630 KB)

Supplementary file2 (JPG 1381 KB)

Supplementary file3 (JPG 1065 KB)

Supplementary file4 (DOCX 2046 KB)

## Data Availability

All *Hepatocystis* and trypanosome sequences of the study are available at GenBank (NCBI) with the accession numbers OR462727-OR462732, OR469745-OR469791.
