# Supplementary figures and images for: First investigation of blood parasites of bats in Burkina Faso detects Hepatocystis parasites and infections with diverse Trypanosoma spp
Source: Parasitol Res. 2023 Oct 17;122(12):3121–9. doi: 10.1007/s00436-023-08002-2 (PMC10667148; doi:10.1007/s00436-023-08002-2)

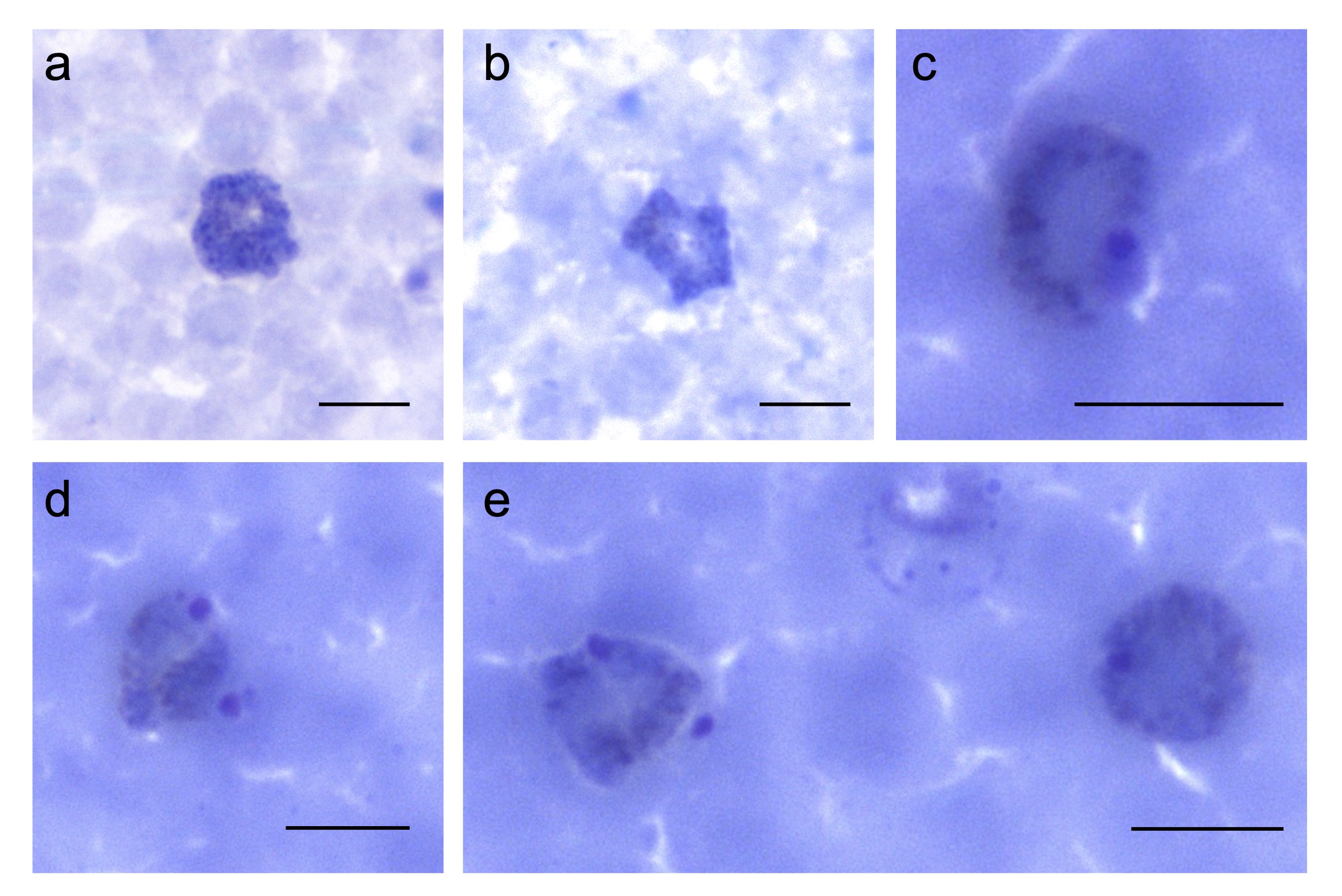

Supplement: Supplementary file 1 — Supplementary file1 (JPG 630 KB) [file 436_2023_8002_MOESM1_ESM.jpg]

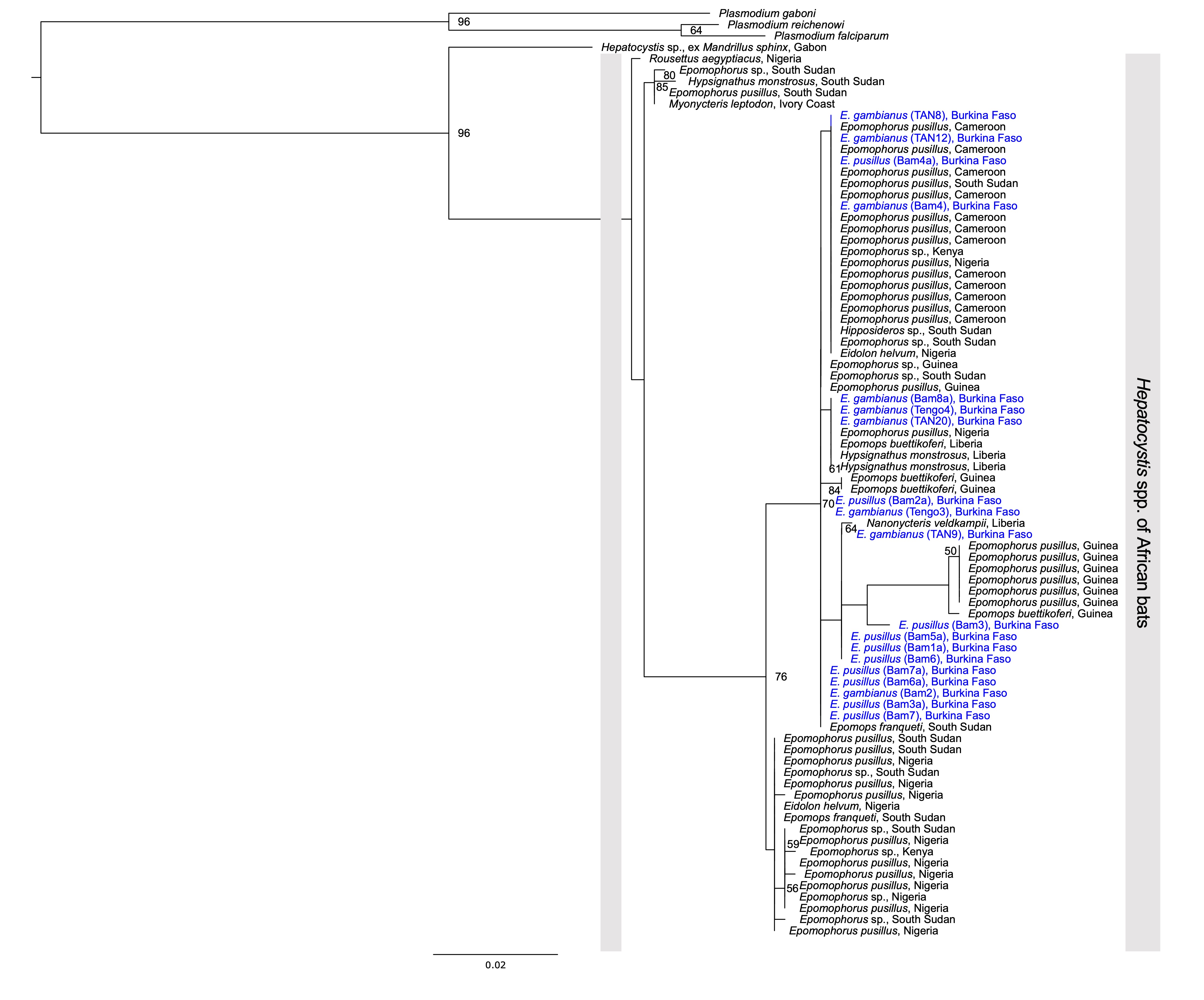

Supplement: Supplementary file 2 — Supplementary file2 (JPG 1381 KB) [file 436_2023_8002_MOESM2_ESM.jpg]

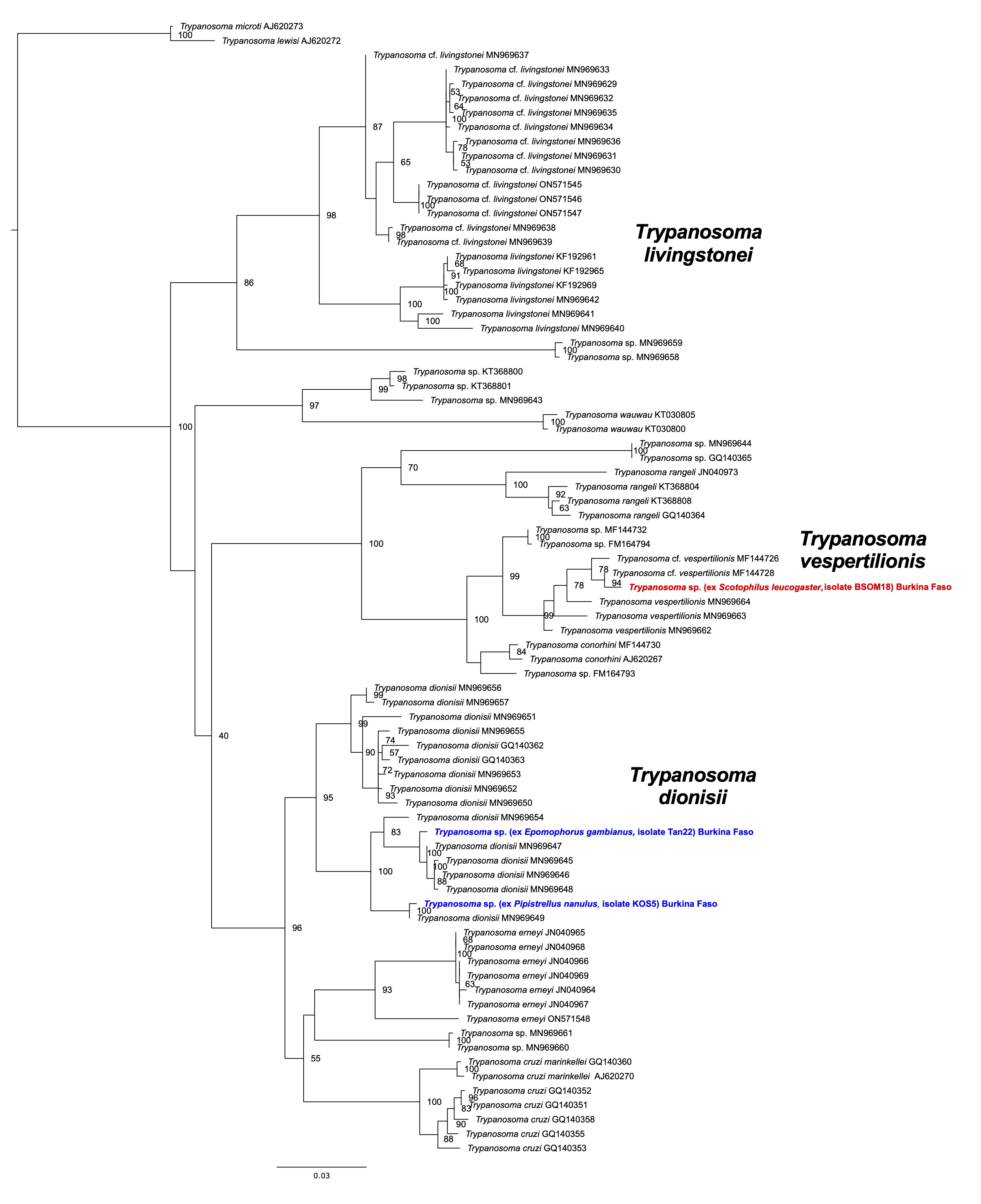

Supplement: Supplementary file 3 — Supplementary file3 (JPG 1065 KB) [file 436_2023_8002_MOESM3_ESM.jpg]
