# Supplementary material for: First investigation of blood parasites of bats in Burkina Faso detects Hepatocystis parasites and infections with diverse Trypanosoma spp
Source: Parasitol Res. 2023 Oct 17;122(12):3121–9. doi: 10.1007/s00436-023-08002-2 (PMC10667148; doi:10.1007/s00436-023-08002-2)
Supplement: Supplementary file 4 — Supplementary file4 (DOCX 2046 KB) [file 436_2023_8002_MOESM4_ESM.docx]

**SUPPLEMENTAL MATERIAL**

First investigation of blood parasites of bats in Burkina Faso detects *Hepatocystis* parasites and infections with diverse *Trypanosoma* spp.

Noel Gabiliga Thiombiano, Magloire Boungou, Bertrand Adéchègoun Mèschac Chabi, Adama Oueda, Oskar Werb, Juliane Schaer

**Content:**

- **Supplemental Tables 1-4**
- **Supplemental Figures 1-3**

**Thiombiano et al., Supplemental Table S1**

**Table S1:** Overview of investigated bat individuals of the study and detected parasite infections

| **Sample ID** | **Bat species** | **Date sampled** | **Season (wet or dry)** | **haemosporidian infection** | ***Trypanosoma* infection** |
| --- | --- | --- | --- | --- | --- |
| BAM2 | *Epomophorus gambianus* | 17.08.2021 | Wet | *Hepatocystis* sp. | negative |
| Bam8a | *Epomophorus gambianus* | 17.08.2021 | Wet | *Hepatocystis* sp. | negative |
| BAZ1 | *Epomophorus gambianus* | 15.08.2021 | Wet | *negative* | negative |
| kam10 | *Epomophorus gambianus* | 12.02.2021 | Dry | *Hepatocystis* sp. | negative |
| kam11 | *Epomophorus gambianus* | 12.02.2021 | Dry | negative | negative |
| kam6 | *Epomophorus gambianus* | 12.02.2021 | Dry | negative | negative |
| kam7 | *Epomophorus gambianus* | 12.02.2021 | Dry | negative | negative |
| kam9 | *Epomophorus gambianus* | 12.02.2021 | Dry | negative | negative |
| Kos3 | *Epomophorus gambianus* | 29.01.2021 | Dry | negative | negative |
| Tan1 | *Epomophorus gambianus* | 21.12.2020 | Wet | *Hepatocystis* sp. | negative |
| Tan21 | *Epomophorus gambianus* | 21.12.2020 | Wet | negative | negative |
| Tan22 | *Epomophorus gambianus* | 21.12.2020 | Wet | negative | *Trypanosoma* cf. *dionsii* |
| Tan23 | *Epomophorus gambianus* | 21.12.2020 | Wet | negative | negative |
| Tan24 | *Epomophorus gambianus* | 21.12.2020 | Wet | negative | negative |
| Tan25 | *Epomophorus gambianus* | 21.12.2020 | Wet | negative | negative |
| Tan26 | *Epomophorus gambianus* | 21.12.2020 | Wet | negative | negative |
| Tan5 | *Epomophorus gambianus* | 21.12.2020 | Wet | negative | negative |
| Tan7 | *Epomophorus gambianus* | 21.12.2020 | Wet | negative | negative |
| Tan9 | *Epomophorus gambianus* | 21.12.2020 | Wet | *Hepatocystis* sp. | negative |
| tengo1 | *Epomophorus gambianus* | 19.02.2021 | Dry | *Hepatocystis* sp. | negative |
| tengo2 | *Epomophorus gambianus* | 19.02.2021 | Dry | negative | negative |
| tengo3 | *Epomophorus gambianus* | 19.02.2021 | Dry | *Hepatocystis* sp. | negative |
| tengo4 | *Epomophorus gambianus* | 19.02.2021 | Dry | *Hepatocystis* sp. | negative |
| BAM4 | *Epomophorus gambianus* | 17.08.2021 | Wet | *Hepatocystis* sp. | negative |
| KOS6 | *Epomophorus gambianus* | 29.01.2021 | Dry | negative | negative |
| Bang6 | *Epomophorus gambianus* | 30.04.2021 | Dry | negative | negative |
| Tan11 | *Epomophorus gambianus* | 21.12.2020 | Wet | *Hepatocystis* sp. | negative |
| Tan12 | *Epomophorus gambianus* | 21.12.2020 | Wet | *Hepatocystis* sp. | negative |
| TAN10 | *Epomophorus gambianus* | 21.12.2020 | Wet | negative | negative |
| TAN20 | *Epomophorus gambianus* | 21.12.2020 | Wet | *Hepatocystis* sp. | negative |
| Tan13 | *Epomophorus gambianus* | 21.12.2020 | Wet | *Hepatocystis* sp. | negative |
| Tan14 | *Epomophorus gambianus* | 21.12.2020 | Wet | *Hepatocystis* sp. | negative |
| TAN16 | *Epomophorus gambianus* | 21.12.2020 | Wet | negative | negative |
| TAN17 | *Epomophorus gambianus* | 21.12.2020 | Wet | negative | negative |
| TAN18 | *Epomophorus gambianus* | 21.12.2020 | Wet | negative | negative |
| TAN3 | *Epomophorus gambianus* | 21.12.2020 | Wet | negative | negative |
| Tan6 | *Epomophorus gambianus* | 21.12.2020 | Wet | *Hepatocystis* sp. | negative |
| Tan8 | *Epomophorus gambianus* | 21.12.2020 | Wet | *Hepatocystis* sp. | negative |
| Bam1a | *Epomophorus pusillus* | 17.08.2021 | Wet | *Hepatocystis* sp. | negative |
| Bam2a | *Epomophorus pusillus* | 17.08.2021 | Wet | *Hepatocystis* sp. | negative |
| BAM3 | *Epomophorus pusillus* | 17.08.2021 | Wet | *Hepatocystis* sp. | negative |
| Bam3a | *Epomophorus pusillus* | 17.08.2021 | Wet | *Hepatocystis* sp. | negative |
| Bam4a | *Epomophorus pusillus* | 17.08.2021 | Wet | *Hepatocystis* sp. | negative |
| Bam5a | *Epomophorus pusillus* | 17.08.2021 | Wet | *Hepatocystis* sp. | negative |
| BAM6' | *Epomophorus pusillus* | 17.08.2021 | Wet | *Hepatocystis* sp. | negative |
| Bam6a | *Epomophorus pusillus* | 17.08.2021 | Wet | *Hepatocystis* sp. | negative |
| BAM7 | *Epomophorus pusillus* | 17.08.2021 | Wet | *Hepatocystis* sp. | negative |
| Bam7a | *Epomophorus pusillus* | 17.08.2021 | Wet | *Hepatocystis* sp. | negative |
| DIE02 | *Hipposideros jonesi* | 18.08.2021 | Wet | negative | negative |
| BSOM20 | *Mops condylurus* | 12.03.2021 | Dry | negative | negative |
| BSOM22 | *Mops condylurus* | 12.03.2021 | Dry | negative | negative |
| BSOM5 | *Mops condylurus* | 12.03.2021 | Dry | negative | negative |
| BSOM10 | *Mops midas* | 12.03.2021 | Dry | negative | negative |
| BSOM12 | *Mops midas* | 12.03.2021 | Dry | negative | negative |
| BSOM13 | *Mops midas* | 12.03.2021 | Dry | negative | negative |
| BSOM14 | *Mops midas* | 12.03.2021 | Dry | negative | negative |
| BSOM15 | *Mops midas* | 12.03.2021 | Dry | negative | negative |
| BSOM16 | *Mops midas* | 12.03.2021 | Dry | negative | negative |
| BSOM17 | *Mops midas* | 12.03.2021 | Dry | negative | negative |
| BSOM19 | *Mops midas* | 12.03.2021 | Dry | negative | negative |
| BSOM23 | *Mops midas* | 12.03.2021 | Dry | negative | negative |
| BSOM24 | *Mops midas* | 12.03.2021 | Dry | negative | negative |
| BSOM3 | *Mops midas* | 12.03.2021 | Dry | negative | negative |
| BSOM4 | *Mops midas* | 12.03.2021 | Dry | negative | negative |
| BSOM6 | *Mops midas* | 12.03.2021 | Dry | negative | negative |
| BSOM7 | *Mops midas* | 12.03.2021 | Dry | negative | negative |
| BSOM8 | *Mops midas* | 12.03.2021 | Dry | negative | negative |
| BSOM9 | *Mops midas* | 12.03.2021 | Dry | negative | negative |
| Baz20 | *Nycteris hispida* | 15.08.2021 | Wet | negative | *Trypanosoma* cf. *livingstonei* |
| kam2 | *Pipistrellus nanulus* | 12.02.2021 | Dry | negative | negative |
| kam4 | *Pipistrellus nanulus* | 12.02.2021 | Dry | negative | negative |
| kam5 | *Pipistrellus nanulus* | 12.02.2021 | Dry | negative | negative |
| Kos4 | *Pipistrellus nanulus* | 29.01.2021 | Dry | negative | negative |
| Kos5 | *Pipistrellus nanulus* | 29.01.2021 | Dry | negative | *Trypanosoma* cf. *dionisii* |
| Tan4 | *Pipistrellus nanulus* | 21.12.2020 | Wet | negative | negative |
| Tan15 | *Pipistrellus nanulus* | 21.12.2020 | Wet | negative | negative |
| BSOM11 | *Pipistrellus nanulus* | 12.03.2021 | Dry | negative | negative |
| BSOM28 | *Pipistrellus nanulus* | 12.03.2021 | Dry | negative | negative |
| Kos12 | *Pipistrellus nanulus* | 29.01.2021 | Dry | negative | negative |
| BAZ13 | *Rhinolophus alcyone* | 15.08.2021 | Wet | negative | negative |
| DIE15 | *Rhinolophus alcyone* | 8.08.2021 | Wet | negative | *Trypanosoma* cf. *livingstonei* |
| DIE16 | *Rhinolophus alcyone* | 8.08.2021 | Wet | negative | *Trypanosoma* cf. *livingstonei* |
| DIE19 | *Rhinolophus alcyone* | 8.08.2021 | Wet | negative | *Trypanosoma* sp. |
| DIE20 | *Rhinolophus alcyone* | 8.08.2021 | Wet | negative | negative |
| DIE21 | *Rhinolophus alcyone* | 8.08.2021 | Wet | negative | negative |
| DIE22 | *Rhinolophus alcyone* | 8.08.2021 | Wet | negative | *Trypanosoma* cf. *livingstonei* |
| DIE23 | *Rhinolophus alcyone* | 8.08.2021 | Wet | negative | negative |
| DIE3 | *Rhinolophus alcyone* | 8.08.2021 | Wet | negative | negative |
| DIE9 | *Rhinolophus alcyone* | 8.08.2021 | Wet | negative | negative |
| DIE13 | *Rhinolophus alcyone* | 8.08.2021 | Wet | negative | negative |
| BAZ10 | *Scotophilus leucogaster* | 15.08.2021 | Wet | negative | negative |
| BAZ11 | *Scotophilus leucogaster* | 15.08.2021 | Wet | negative | *Trypanosoma* cf. *vespertilionis* |
| BAZ17 | *Scotophilus leucogaster* | 15.08.2021 | Wet | negative | negative |
| BAZ18 | *Scotophilus leucogaster* | 15.08.2021 | Wet | negative | negative |
| BAZ19 | *Scotophilus leucogaster* | 15.08.2021 | Wet | negative | negative |
| BAZ21 | *Scotophilus leucogaster* | 15.08.2021 | Wet | negative | *Trypanosoma* cf. *vespertilionis* |
| BAZ22 | *Scotophilus leucogaster* | 15.08.2021 | Wet | negative | negative |
| BAZ23 | *Scotophilus leucogaster* | 15.08.2021 | Wet | negative | negative |
| BAZ24 | *Scotophilus leucogaster* | 15.08.2021 | Wet | negative | negative |
| BAZ5 | *Scotophilus leucogaster* | 15.08.2021 | Wet | negative | *Trypanosoma* cf. *vespertilionis* |
| BAZ6 | *Scotophilus leucogaster* | 15.08.2021 | Wet | negative | *Trypanosoma* cf. *vespertilionis* |
| BAZ8 | *Scotophilus leucogaster* | 15.08.2021 | Wet | negative | negative |
| BAZ9 | *Scotophilus leucogaster* | 15.08.2021 | Wet | negative | negative |
| KIR1 | *Scotophilus leucogaster* | 16.08.2021 | Wet | negative | negative |
| KIR10 | *Scotophilus leucogaster* | 16.08.2021 | Wet | negative | negative |
| KIR11 | *Scotophilus leucogaster* | 16.08.2021 | Wet | negative | negative |
| KIR15 | *Scotophilus leucogaster* | 16.08.2021 | Wet | negative | negative |
| KIR2 | *Scotophilus leucogaster* | 16.08.2021 | Wet | negative | negative |
| KIR3 | *Scotophilus leucogaster* | 16.08.2021 | Wet | negative | negative |
| KIR4 | *Scotophilus leucogaster* | 16.08.2021 | Wet | negative | negative |
| KIR5 | *Scotophilus leucogaster* | 16.08.2021 | Wet | negative | negative |
| KIR6 | *Scotophilus leucogaster* | 16.08.2021 | Wet | negative | negative |
| KIR7 | *Scotophilus leucogaster* | 16.08.2021 | Wet | negative | negative |
| KIR8 | *Scotophilus leucogaster* | 16.08.2021 | Wet | negative | negative |
| KIR9 | *Scotophilus leucogaster* | 16.08.2021 | Wet | negative | negative |
| BSOM1 | *Scotophilus leucogaster* | 12.03.2021 | Dry | negative | negative |
| BSOM18 | *Scotophilus leucogaster* | 12.03.2021 | Dry | negative | *Trypanosoma* cf. *vespertilionis* |
| BSOM2 | *Scotophilus leucogaster* | 12.03.2021 | Dry | negative | negative |
| BSOM21 | *Scotophilus leucogaster* | 12.03.2021 | Dry | negative | negative |

**Thiombiano et al., Supplemental Table S2**

**Table S2:** Nucleotide primers used for haemosporidian and trypanosome parasite screening and sequencing

| **Gene** | **Primer name** | **Sequence (5´- 3´)** | **Reference** |
| --- | --- | --- | --- |
| ***cytb*** | Hep-F3 | CTTACCTTGGGGACAAATGAGTTATT | Schaer et al., 2013 |
|  | Hep-R3 | CTCTAGCACCAAATGTCATTTTAAATTG | Schaer et al., 2013 |
|  | DW2 | TAATGCCTAGACGTATTCCTGATTATCCAG | Perkins & Schall, 2002 |
|  | DW4 | TGTTTGCTTGGGAGCTGTAATCATAATGTG | Perkins & Schall, 2002 |
|  | 3932-F | GGGTTATGTATTACCTTGGGGTC | Perkins and Schall, 2002 |
|  | 3932-R | GACCCCAAGGTAATACATAACCC | Perkins & Schall, 2002 |
| ***cox1*** | Cox1-F | CTATTTATGGTTTTCATTTTTATTTGGTA | Martinsen et al., 2008 |
|  | Cox1-R | AGGAATACGTCTAGGCATTACATTAAATCC | Martinsen et al., 2008 |
|  | Cox-in-F | ATGATATTTACARTTCAYGGWATTATTATG | Martinsen et al., 2008 |
|  | Cox-in-R | GTATTTTCTCGTAATGTTTTACCAAAGAA | Martinsen et al., 2008 |
|  | Cox-mid-F | TTATTCTGGTTTTTTGGTCATCCAG | Martinsen et al., 2008 |
|  | Cox-mid-R | CTGGATGACCAAAAAACCAGAATAA | Martinsen et al., 2008 |
| ***ef2*** | EF2-F | GTTCGTGAGATCATGAACAAAAC | Schaer et al., 2013 |
|  | EF2-R | CCTTGTAAACCAGAACCAAA | Schaer et al., 2013 |
| ***18S rRNA*** | TRY927F | GAAACAAGAAACACGGGAG | Noyes et al., 1999 |
|  | TRY927R | CTACTGGGCAGCTTGGA | Noyes et al., 1999 |
|  | SSU561F | TGGGATAACAAAGGAGCA | Noyes et al., 1999 |
|  | SSU561R | CTGAGACTGTAACCTCAAAGC | Noyes et al., 1999 |
| ***gGAPDH*** | G5 | ACMAGRTCCACCACRCGGTG | Hamilton et al., 2004 |
|  | G3 | TTYGCCGYATYGGYCGCATGG | Hamilton et al., 2004 |
|  | G1 | CGCGGATCCASGGYCTYMTCGGBAMKGAGAT | Hamilton et al., 2004 |
|  | G4A | GTTYTGCAGSGTCGCCTTGG | Hamilton et al., 2004 |

*cytb*, cytochrome b; *cox1*, cytochrome oxidase I; *ef2*, nuclear elongation factor 2; *18S rRNA,* SSU rRNA, a component of the eukaryotic ribosomal small subunit; *gGAPDH,* Glyceraldehyde-3-phosphate dehydrogenase

Hamilton, P.B., Stevens, J.R., Gaunt, M.W., Gidley, J., Gibson, W.C. 2004. Trypanosomes are monophyletic: evidence from genes for glyceraldehyde phosphate dehydrogenase and small subunit ribosomal RNA. Int. J. Parasitol. 34, 1393–1404.

Martinsen, E.S., Perkins, S.L., Schall, J., 2008. A three-genome phylogeny of malaria parasites (*Plasmodium* and closely related genera): evolution of life-history traits and host switches. Mol. Phylogenet. Evol. 47, 261–273.

Noyes, H.A., Stevens, J.R., Teixeira, M., Phelan, J., Holz, P. 1999. A nested PCR for the ssrRNA gene detects *Trypanosoma binneyi* in the *Platypus* and *Trypanosoma* sp. in wombats and kangaroos in Australia. Int. J. Parasitol. 29, 331–39.

Perkins, S.L., Schall, J., 2002. A molecular phylogeny of malarial parasites recovered from cytochrome *b* gene sequences. J. Parasitol. 88, 972–978.

Schaer, J., Perkins, S.L., Decher, J., Leendertz, F.H., Fahr, J., Weber, N., Matuschewski, K., 2013. High diversity of West African bat malaria parasites and a tight link with rodent *Plasmodium* taxa. Proc. Natl. Acad. Sci. USA. 110, 17415-17419.

**Thiombiano et al., Supplemental Table S3**

**Table S3:** GenBank accession numbers for phylogenetic analysis of *Hepatocystis* parasites (samples from this study highlighted in bold)

| **Parasite (host group)** | **Sample (host species)** | ***cytb*** | ***Cox1*** | ***Ef2*** |
| --- | --- | --- | --- | --- |
| *Leucocytozoon* (Aves) | *Leucocytozoon* sp. (2109) | EU254518 | EU254563 | -- |
|  | *Leucocytozoon* sp. (2208) | EU254520 | EU254565 | -- |
|  | *Leucocytozoon* sp. (P157) | EU254519 | EU254564 | -- |
| *Haemoproteus* (Aves) | *Haemoproteus columbae* (2111) | EU254548 | -- | -- |
|  | *Haemoproteus columbae* (2146) | EU254553 | -- | -- |
|  | *Haemoproteus columbae* | FJ168562 | FJ168562 | -- |
| *Parahaemoproteus* (Aves) | *Parahaemoproteus coatneyi* | EU254550 | EU254595 | -- |
|  | *Parahaemoproteus belopolskyi* | DQ451408 | EU254603 | -- |
| *Plasmodium* (Primates) | *Plasmodium falciparum* | DQ642845 | M76611 | DQ642846 |
|  | *Plasmodium gaboni* | FJ895307 | FJ895307 | -- |
|  | *Plasmodium reichenowi* | AJ251941 | AJ251941 | -- |
|  | *Plasmodium* sp. (ex-*Pan troglodytes*) | HM235391 | HM235391 | -- |
|  | *Plasmodium* sp. (ex *Gorilla gorilla)* | HM235288 | HM235308 | -- |
|  | *Plasmodium knowlesi* | AF069621 | EU880499 | XM002260326 |
|  | *Plasmodium vivax* | KY923424 | KY92342 | XM_00161582 |
|  | *Plasmodium ovale* | AF069625 | JF894415 | -- |
|  | *Plasmodium malariae* | AF069624 | AB489193 | -- |
|  | *Plasmodium cynomolgi* | AF069616 | MN372343 | -- |
|  | *Plasmodium inui* | AF069617 | AB354572 | -- |
| *Plasmodium* (Rodentia) | *Plasmodium berghei* | DQ414645 | DQ414589 | -- |
|  | *Plasmodium chabaudi* | DQ414649 | DQ414593 | XM_736543 |
|  | *Plasmodium vinckei* | DQ414651 | DQ414596 | -- |
|  | *Plasmodium yoelii* | AY099051 | DQ414605 | LM993667 |
| *Plasmodium* (Chiroptera) | *Plasmodium cyclopsi* | KF159710 | KF159788 | KF159729 |
|  | *Plasmodium voltaicum* | KF159671 | KF159792 | -- |
| *Polychromophilus* (Chiroptera) | *Polychromophilus* sp. (ex *Miniopterus villiersii*) | KF159681 | KF159796 | KF159731 |
|  | *Polychromophilus melanipherus* | JN990709 | -- | -- |
|  | *Polychromophilus murinus* | HM055583 | -- | -- |
|  | *Polychromophilus* sp. (ex *Pipistrellus grandidieri*) | KF159714 | KF159797 | KF159742 |
| *Hepatocystis* (Chiroptera) | *Hepatocystis* sp. (ex *E. pusillus,* Guinea) | KF159683 | KF159801 | KF159744 |
|  | *Hepatocystis* sp. (ex *E. pusillus,* Guinea) | KF159680 | KF159778 | KF159723 |
|  | *Hepatocystis* sp. (ex *E. pusillus,* Guinea) | KF159693 | KF159773 | KF159746 |
|  | *Hepatocystis* sp. (ex *E. pusillus,* Guinea) | KF159704 | KF159775 | KF159753 |
|  | *Hepatocystis* sp. (ex *E. pusillus,* Guinea) | KF159683 | KF159801 | KF159744 |
|  | *Hepatocystis* sp. (ex *E. pusillus,* Cameroon) | MZ460922 | --- | --- |
|  | *Hepatocystis* sp. (ex *E. pusillus,* Cameroon) | MZ460918 | --- | --- |
|  | *Hepatocystis* sp. (ex *E. pusillus,* Cameroon) | MZ460915 | --- | --- |
|  | *Hepatocystis* sp. (ex *E. pusillus,* Nigeria) | MK634490 | --- | MK634512 |
|  | *Hepatocystis* sp. (ex *E. pusillus,* Nigeria) | MK634507 | --- | MK634523 |
|  | *Hepatocystis* sp. (ex *E. pusillus,* Nigeria) | MK634505 | --- | MK634521 |
|  | *Hepatocystis* sp. (ex *E. pusillus,* Nigeria) | MK634501 | --- | MK634517 |
|  | *Hepatocystis* sp. (ex *E. pusillus,* Nigeria) | MK634496 | --- | MK634515 |
|  | *Hepatocystis* sp. (ex *E. pusillus,* Nigeria) | MK634503 | --- | MK634519 |
|  | *Hepatocystis* sp. (ex *E. pusillus,* Nigeria) | MK634487 | --- | MK634509 |
|  | *Hepatocystis* sp. (ex *E. pusillus,* Nigeria) | MK634497 | --- | MK634516 |
|  | *Hepatocystis* sp. (ex *E. pusillus,* Nigeria) | MK634489 | --- | MK634511 |
|  | *Hepatocystis* sp. (ex *E. pusillus,* Nigeria) | MK634506 | --- | MK634522 |
|  | *Hepatocystis* sp. (ex *E. pusillus,* Nigeria) | MK634488 | --- | MK634510 |
|  | *Hepatocystis* sp. (ex *E. pusillus,* South Sudan) | KY753527 | KY753543 | KY753591 |
|  | *Hepatocystis* sp. (ex *E. pusillus,* South Sudan) | KY753525 | KY753541 | KY753589 |
|  | *Hepatocystis* sp. (ex *Epom.* sp*.,* Kenya) | KY753518 | KY753536 | KY753583 |
|  | *Hepatocystis* sp. (ex *Epom.* sp*.,* Kenya) | KY753519 | --- | KY753584 |
|  | *Hepatocystis* sp. (ex *Epom.* sp.*,* South Sudan) | KY753506 | KY753530 | KY753571 |
|  | *Hepatocystis* sp. (ex *Epom.* sp.*,* South Sudan) | KY753507 | KY753531 | KY753572 |
|  | *Hepatocystis* sp. (ex *Epom.* sp.*,* South Sudan) | KY753513 | KY753535 | KY753578 |
|  | *Hepatocystis* sp. (ex *Epom.* sp.*,* South Sudan) | KY753510 | KY753534 | KY753575 |
|  | *Hepatocystis* sp. (ex *Epom.* sp.*,* South Sudan) | KY753516 | --- | KY753581 |
|  | *Hepatocystis* sp. (ex *E. buettikoferi*, Guinea) | KF159706 | KF159779 | KF159757 |
|  | *Hepatocystis* sp. (ex *E. buettikoferi*, Guinea) | KF159703 | KF159790 | --- |
|  | *Hepatocystis* sp. (ex *E. franqueti*, Uganda) | KT750344 | KT750505 | --- |
|  | *Hepatocystis* sp. (ex *E. franqueti*, Uganda) | KT750356 | KT750527 | --- |
|  | *Hepatocystis* sp. (ex *E. franqueti*, Uganda) | KT750351 | KT750526 | --- |
|  | *Hepatocystis* sp. (ex *E. franqueti*, Uganda) | KT750353 | KT750524 | --- |
|  | *Hepatocystis* sp. (ex *E. franqueti*, South Sudan) | KY753503 | KY753528 | KY753568 |
|  | *Hepatocystis* sp. (ex *E. franqueti*, South Sudan) | KY753504 | --- | KY753569 |
|  | *Hepatocystis* sp. (ex *Hipposideros* sp.*,* South Sudan) | KY753520 | --- | --- |
|  | *Hepatocystis* sp. (ex *H. monstrosus,* Liberia) | KF159689 | KF159799 | KF159734 |
|  | *Hepatocystis* sp. (ex *H. monstrosus,* Liberia) | KF159712 | --- | --- |
|  | *Hepatocystis* sp. (ex *H. monstrosus,* South Sudan) | KY753521 | KY753537 | KY753585 |
|  | *Hepatocystis* sp. (ex *M. leptodon,* Ivory Coast) | KF188066 | KF188069 | KF188071 |
|  | *Hepatocystis* sp. (ex *M. leptodon,* Liberia) | KF159705 | --- | KF159754 |
|  | *Hepatocystis* sp. (ex *M. torquata,* Uganda) | KT750356 | KT750527 | --- |
|  | *Hepatocystis* sp. (ex *M. torquata,* Uganda) | KT750357 | KT750521 | --- |
|  | *Hepatocystis* sp. (ex *M. torquata,* Uganda) | KT750342 | KT750534 | --- |
|  | *Hepatocystis* sp. (ex *N. veldkampii,* Guinea) | KF159698 | KF159786 | KF159749 |
|  | *Hepatocystis* sp. (ex *N. veldkampii,* Guinea) | EU254528 | EU254571 | --- |
|  | *Hepatocystis* sp. (ex *N. veldkampii,* Liberia) | KF159698 | KF159786 | KF159749 |
|  | *Hepatocystis* sp. (ex *R. aegyptiacus,* Nigeria) | MK634508 | --- | MK634524 |
|  | *Hepatocystis* sp. (*ex Eidolon helvum,* Gabon) | MG602649 | --- | --- |
|  | *Hepatocystis* sp. (ex *Eidolon helvum* Nigeria) | ON494563 | --- | --- |
|  | *Hepatocystis* sp. (ex *Eidolon helvum*, Nigeria) | ON494561 | --- | --- |
|  | *Hepatocystis* sp. (ex *Eidolon helvum,* Nigeria) | ON494560 | --- | --- |
|  | *Hepatocystis* sp. (ex *Eidolon helvum*, Nigeria) | ON494562 | --- | --- |
| ***Hepatocystis* (Chiroptera)**  **Burkina Faso** | ***Hepatocystis* sp. (ex *E. pusillus*, isolate Bam1a)*** | **OR469748** | **OR469767** | **OR469780** |
|  | ***Hepatocystis* sp. (ex *E. pusillus*, isolate Bam3)*** | **OR469764** | **OR469768** | **OR469781** |
|  | ***Hepatocystis* sp. (ex *E. pusillus*, isolate Bam3a)*** | **OR469763** | **OR469769** | **OR469782** |
|  | ***Hepatocystis* sp. (ex *E. pusillus*, isolate Bam5a)*** | **OR469760** | **OR469770** | **OR469783** |
|  | ***Hepatocystis* sp. (ex *E. pusillus*, isolate Bam6a)*** | **OR469758** | **OR469771** | **OR469784** |
|  | ***Hepatocystis* sp. (ex *E. pusillus*, isolate Bam7)*** | **OR469757** | **OR469772** | **OR469785** |
|  | ***Hepatocystis* sp. (ex *E. pusillus*, isolate Bam7a)*** | **OR469756** | **OR469773** | **OR469786** |
|  | ***Hepatocystis* sp. (ex *E. pusillus*, isolate Bam2a)** | **OR469765** | **---** | **---** |
|  | ***Hepatocystis* sp. (ex *E. pusillus*, isolate Bam4a)** | **OR469761** | **---** | **---** |
|  | ***Hepatocystis* sp. (ex *E. pusillus*, isolate Bam6)** | **OR469759** | **---** | **---** |
|  | ***Hepatocystis* sp. (ex *E. gambianus*, isolate Bam2)** | **OR469766** | **---** | **---** |
|  | ***Hepatocystis* sp. (ex *E. gambianus*, isolate Bam4)** | **OR469762** | **---** | **---** |
|  | ***Hepatocystis* sp. (ex *E. gambianus*, isolate Bam8a)** | **OR469755** | **---** | **---** |
|  | ***Hepatocystis* sp. (ex *E. gambianus*, isolate Tan1)*** | **--** | **OR469775** | **OR469788** |
|  | ***Hepatocystis* sp. (ex *E. gambianus*, isolate Tan6)*** | **--** | **OR469776** | **OR469789** |
|  | ***Hepatocystis* sp. (ex *E. gambianus*, isolate Tan8)*** | **OR469750** | **---** | **OR469790** |
|  | ***Hepatocystis* sp. (ex *E. gambianus*, isolate Tan9)*** | **OR469749** | **---** | **OR469791** |
|  | ***Hepatocystis* sp. (ex *E. gambianus*, isolate Tan11)** | **--** | **OR469777^1^** | **--** |
|  | ***Hepatocystis* sp. (ex *E. gambianus*, isolate Tan12)*** | **OR469751** | **---** | **---** |
|  | ***Hepatocystis* sp. (ex *E. gambianus*, isolate Tan13)** | **--** | **OR469778^2^** | **--** |
|  | ***Hepatocystis* sp. (ex *E. gambianus*, isolate Tan14)** | **--** | **OR469779^3^** | **--** |
|  | ***Hepatocystis* sp. (ex *E. gambianus*, isolate Tan20)*** | **OR469752** | **---** | **---** |
|  | ***Hepatocystis* sp. (ex *E. gambianus*, isolate Tengo1)** | **--** | **NA**** | **--** |
|  | ***Hepatocystis* sp. (ex *E. gambianus*, isolate Tengo3)*** | **OR469753** | **---** | **---** |
|  | ***Hepatocystis* sp. (ex *E. gambianus*, isolate Tengo4)*** | **OR469754** | **---** | **---** |
|  | ***Hepatocystis* sp. (ex *E. gambianus*, isolate Kam10)*** | **---** | **OR469774** | **OR469787** |
| *Hepatocystis* (Primates) | *Hepatocystis* sp. (ex *C. nictitans,* Cameroon) | JQ070956 | --- | --- |
|  | *Hepatocystis* sp. (ex *C. nictitans,* Cameroon) | JQ070816 | --- | --- |
|  | *Hepatocystis* sp. (ex *C. cephus,* Gabon) | JF923760 | --- | --- |
|  | *Hepatocystis* sp. (ex *C. cephus,* Gabon) | JF923758 | --- | --- |
|  | *Hepatocystis* sp. (ex *P. nubensis,* Ethiopia) | AF069626 | --- | --- |
|  | *Hepatocystis* sp. (ex monkey*,* Uganda) | KC262867 | --- | --- |
|  | *Hepatocystis* sp. (ex monkey*,* Uganda) | GU945305 | --- | --- |
|  | *Hepatocystis* sp. (ex *P. badius,* Uganda) | KC262797 | --- | --- |
|  | *Hepatocystis* sp. (ex *P. badius,* Uganda) | KC262824 | --- | --- |
|  | *Hepatocystis* sp. (ex *M. sphinx,* Gabon) | JF923759 | --- | --- |
|  | *Hepatocystis* sp. (ex *M. talapoin,* Gabon) | JF923757 | --- | --- |
|  | *Hepatocystis* sp. (ex *Macaque,* Southeast Asia) | GU929945 | --- | --- |
|  | *Hepatocystis* sp. (ex *Macaque,* Southeast Asia) | GU929944 | --- | --- |
|  | *Hepatocystis* sp. (ex *Macaque,* Thailand) | EU400409 | --- | --- |
|  | *Hepatocystis* sp. (ex *Macaque,* Myanmar) | HQ605040 | --- | --- |

*included in phylogenetic analysis of concatenated dataset (**Fig. 2**) ; **sequence of low quality that shares highest identity with *Hepatocystis* sp. in the BLASTn search. ^1^in the cox1 (437bp) alignment: the sequence of TAN11 is identical to Bam3a, ^2^sequence of TAN13 is two bases different to Bam3a/TAN11, ^3^sequence of TAN14 is identical to TAN6

| **Thiombiano et al., Supplemental Table S4**  **Table S4**: GenBank accession numbers for *Trypanosoma* parasite sequences of the study   \| **Sample ID** \| **Bat species** \| ***Trypanosoma* species** \| **18S rRNA** \| **gGAPDH** \| \| --- \| --- \| --- \| --- \| --- \| \| Tan22 \| *Epomophorus gambianus* \| *T. dionsii* \| OR462727 \| OR469745 \| \| Baz20 \| *Nycteris hispida* \| *T.* cf. *livingstonei* \| OR462728 \| - \| \| Kos5 \| *Pipistrellus nanulus* \| *T. dionsii* \| OR462729 \| OR469746 \| \| DIE15 \| *Rhinolophus alcyone* \| *T.* *livingstonei* \| OR462730* \| - \| \| DIE19 \| *Rhinolophus alcyone* \| *T.* sp. \| OR462731 \| - \| \| BAZ21 \| *Scotophilus leucogaster* \| *T.* *vespertilionis* \| OR462732** \| - \| \| BSOM18 \| *Scotophilus leucogaster* \| *T.* cf. *vespertilionis* \| - \| OR469747 \|   *3/4 trypanosome samples of *R. alcyone* share identical 18S rRNA sequences and thus represent one haplotype (isolates DIE15, DIE16, DIE22). The representative sample for this haplotype (isolate DIE15) was included in the 18S rRNA phylogenetic analysis; **the 18S rRNA trypanosome sequences of four samples of *S. leucogaster* (isolates BAZ5, BAZ6, BAZ11, BAZ21) represent one haplotype and one representative sample (isolate BAZ21) was included in the 18S rRNA phylogenetic analysis; - = sequence could not be successfully amplified |
| --- | --- | --- | --- | --- | --- | --- | --- | --- | --- | --- | --- | --- | --- | --- | --- | --- | --- | --- | --- | --- | --- | --- | --- | --- | --- | --- | --- | --- | --- | --- | --- | --- | --- | --- | --- | --- | --- | --- | --- | --- |

**Thiombiano et al., Supplemental Figure S1**

**
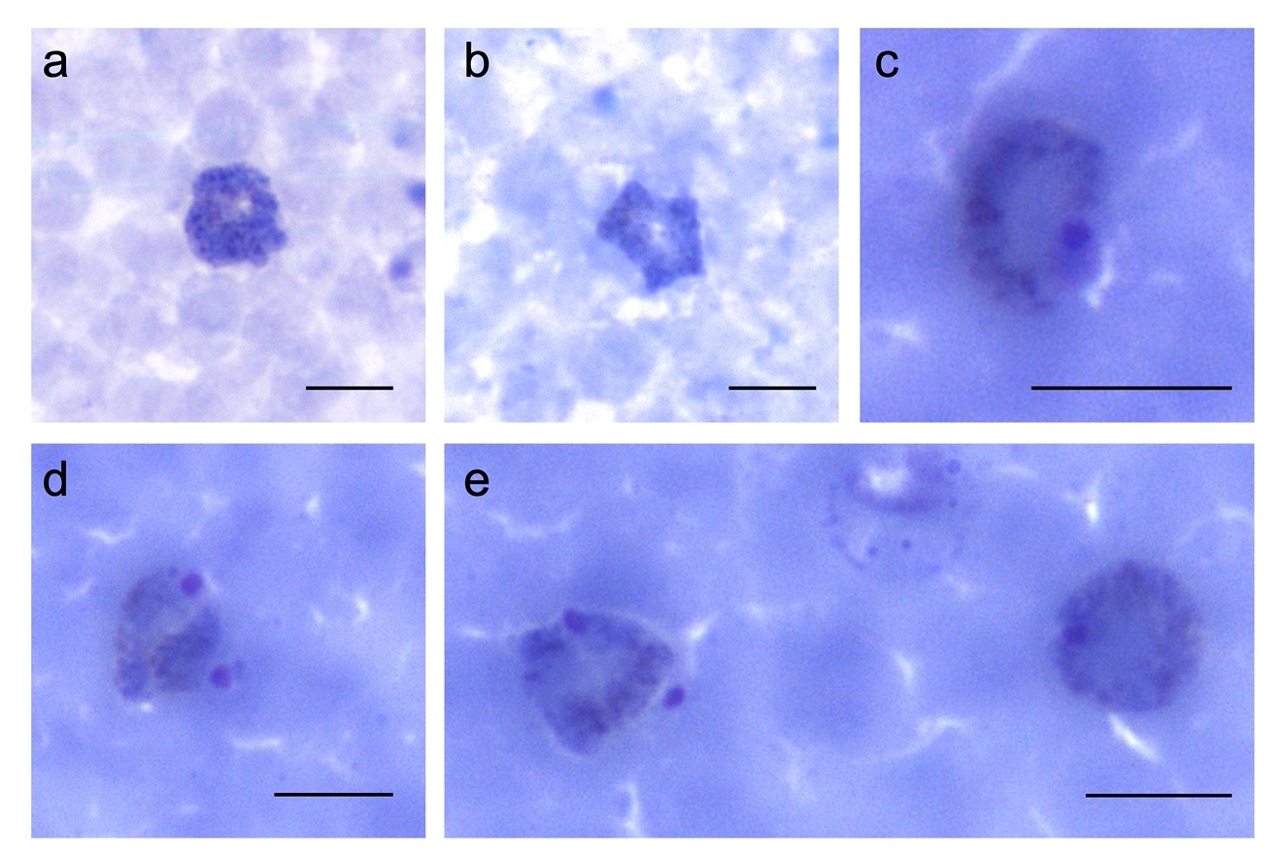
**

**Figure S1.** Representative micrographs of Giemsa-stained thin blood films of mature gametocytes of *Hepatocystis* parasites of *Epomophorus pusillus* bats. The quality of the blood smears did not permit a detailed evaluation of the morphology of the gametocyte blood stages of the *Hepatocystis* parasites. The detected blood stages were limited to mature gametocyte stages and hemozoin pigment was present in all parasite cells in the infected erythrocytes. Deformations of the erythrocyte host cells due to large parasitic vacuoles were common (e.g., in pictures **a** and **b**), resulting in amoeboid cell forms. Overall, the morphology of the detected blood stages corresponds to the descriptions of *Hepatocystis epomophori* parasites of epauletted fruit bats from other African countries**. (a,b)** macrogametocytes with blue-stained cytoplasm in sample numbers Bam7 and Bam3. **(c,d,e)** microgametocytes with visible nuclei, biscuit-colored cytoplasm and vacuoles in sample number Bam4. Note the double-infected erythrocyte in **(e)** on the left. Bars = 5 μm*.*

**Thiombiano et al., Supplemental Figure S2**

**
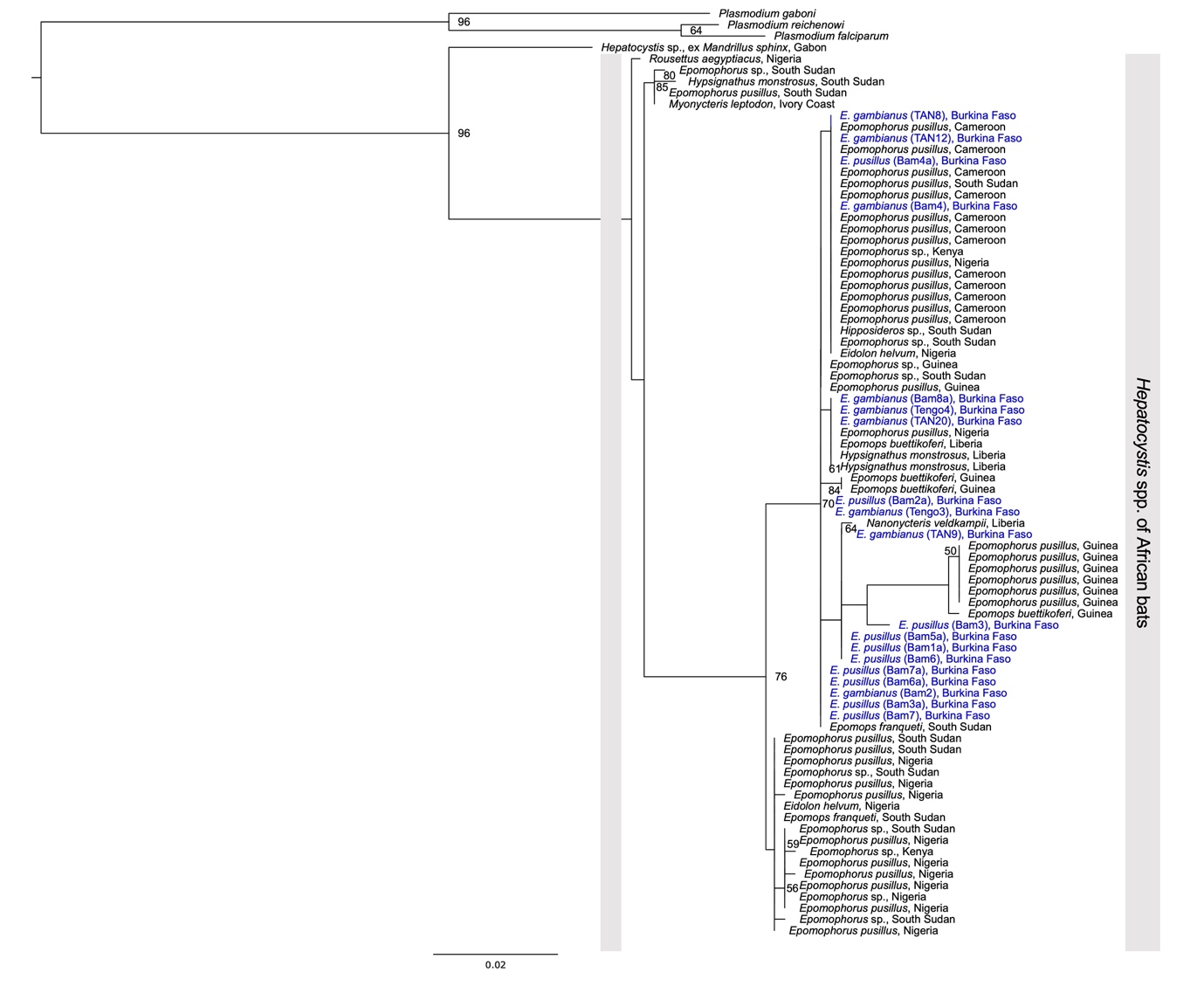
**

**Fig. S2. Maximum likelihood analysis of *Hepatocystis* parasites in African bat species**. The analysis is based on the parasites mitochondrial gene *cytb* (531nt). *Plasmodium falciparum* was used as outgroup taxon. The phylogenetic analysis recovered the *Hepatocystis* sequences from *E. pusillus* and *E. gambianus* of this study (*cytb* sequences could successfully be generated for 18 of 26 samples) within the African bat *Hepatocystis* clade (as also shown in **Fig. 2**). No strict clustering of African bat *Hepatocystis* sequences according to country or host species is apparent. Sequences of the study from Burkina Faso are highlighted in blue. Numbers at nodes are ML bootstrap values (> 50) using 1,000 replicates.

**Thiombiano et al., Supplemental Figure S3**

**Figure S3.** Phylogeny of the *Trypanosoma* parasites inferred by maximum likelihood analysis of the *gGAPDH* gene (894 bp) using GTR + I + G (with 10,000 thorough bootstrapping) and the outgroup taxon *Trypanosoma lewisi.* The parasites isolated from *S. leucogaster* (highlighted in bold red) group within a clade of *T. vespertilionis* and *T.* cf. *vespertilionis* parasites. The parasites of *E. gambianus* and *P. nanulus* of this study (highlighted in bold blue) most likely represent the species *T. dionsii.*
